# Folate deficient tumor microenvironment promotes epithelial-to-mesenchymal transition and cancer stem-like phenotypes

**DOI:** 10.18632/oncotarget.8910

**Published:** 2016-04-22

**Authors:** Yen-Hao Su, Wen-Chien Huang, Tse-Hung Huang, Yan-Jiun Huang, Yu-Kai Sue, Thanh-Tuan Huynh, Michael Hsiao, Tsan-Zon Liu, Alexander TH Wu, Chien-Min Lin

**Affiliations:** ^1^ Department of Surgery, Division of General Surgery, Shuang Ho Hospital, Taipei Medical University, Taipei, Taiwan; ^2^ Institute of Traditional Medicine, School of Medicine, National Yang Ming University, Taipei, Taiwan; ^3^ Department of Thoracic Surgery, Mackay Memorial Hospital, Taipei, Taiwan; ^4^ Department of Traditional Chinese Medicine, Chang Gung Memorial Hospital, Keelung, Taiwan; ^5^ Graduate Institute of Clinical Medicine Sciences, Chang Gung University, Taoyuan, Taiwan; ^6^ Department of Surgery, Division of General Surgery, Taipei Medical University Hospital, Taipei, Taiwan; ^7^ Department of Neurosurgery, Taipei Medical University-Shuang Ho Hospital, Taipei, Taiwan; ^8^ Center for Molecular Biomedicine, University of Medicine and Pharmacy, HoChiMinh City, Viet Nam; ^9^ Genomics Research Center, Academia Sinica, Nankang, Taipei, Taiwan; ^10^ Translational Research Laboratory, Cancer Center, Taipei Medical University and Hospital, Taipei, Taiwan; ^11^ The Ph.D. Program for Translational Medicine, College of Science and Technology, Taipei Medical University, Taipei, Taiwan; ^12^ Department of Neurology, School of Medicine, College of Medicine, Taipei Medical University

**Keywords:** folate deficiency, oxidative/nitrosative stress, epithelial-mesenchymal transition, cancer stem-like cells, miR-22

## Abstract

Clinically, serum level of folate has been negatively correlated to the stage and progression of liver cancer. Nevertheless, the functional consequence of folate deficiency (FD) in malignancy has not been fully investigated. Human hepatocellular carcinoma (HCC) cells (as study model) and other cancer types such as lung and glioma were cultured under folate deficient (FD) and folate complete (FD) conditions. Molecular characterization including intracellular ROS/RNS (reactive oxygen/nitrogen species), viability, colony formation, cancer stem-like cell (CSC) phenotype analyses were performed. *In vivo* tumorigenesis under FD and FC conditions were also examined. FD induced a significant increase in ROS and RNS, suppressing proliferative ability but inducing metastatic potential. Mesenchymal markers such as Snail, ZEB2, and Vimentin were significantly up-regulated while E-cadherin down-regulated. Importantly, CSC markers such as Oct4, β-catenin, CD133 were induced while PRRX1 decreased under FD condition. Furthermore, FD-conditioned HCC cells showed a decreased miR-22 level, leading to the increased expression of its target genes including HDAC4, ZEB2 and Oct4. Finally, xenograft mouse model demonstrated that FD diet promoted tumorigenesis and metastasis as compared to their FC counterparts. Our data provides rationales for the consideration of folate supplement as a metastasis preventive measure.

## INTRODUCTION

Liver cancer ranks the fifth most common cancer globally and the high incidence of distant metastasis makes it the third most common cause of cancer mortality [1]. Despite extensive research into the biology of liver cancer progression, the underlying molecular explanations remains unresolved, in particular, the mechanisms responsible for distant metastasis. Accumulating evidence has shown that oxidative stress (OS) which has long been thought to contribute in the initiation and malignant transformation during carcinogenesis [2], also involves in promoting cancer metastasis [3, 4]. Liver is the major organ prone to OS from noxious substances such as chemicals/drugs, alcohol, environmental pollutants and deficiency in micronutrients. One of the micronutrients, folate (or folic acid), plays an essential role in liver physiology. Folate, a water-soluble B vitamin found abundantly in fresh fruits and leafy green vegetables, has been targeted as a potential chemo-preventive agent [5]. Folate functions to provide one-carbon groups for DNA synthesis/replication, methylation and epigenetic control of gene expression [6–8]. Because liver is the major site for folate storage and susceptible to folate deficiency (FD), deprivation in this micronutrient may contribute to chromosomal breaks and deleterious alterations in gene expression leading to genetic instability and carcinogenesis [9–11]. For instance, methyl-deficient diet (lack of folate, choline and methionine) resulted in altered methylation patterns in hepatic p53 gene which was implicated in the increased risks for hepatocarcinogenesis and/or progression of liver cancer [12–16]. A previous clinical study has established an inverse correlation between the serum folate level and tumor size, multiplicity and metastasis; when disease progression was categorized into stages I to IV, serum folate decreased as disease stage progressed [17]. Another larger cohort study demonstrated that higher folate level in red blood cells was associated with reduced risk of hepatocarcinogenesis [18]. Moreover, it has been suggested that folate may play important roles in preventing tumorigenesis in different cancer types [19–21]. These findings have implicated the importance of folate status in the carcinogenesis of hepatocellular carcinoma and others. However, mechanistic explanations linking FD to the promotion of distant metastasis of liver cancer cells remains to be elucidated.

Cancer stem cells (CSCs) have been shown to a key contributor to the aggressive phenotypes including treatment resistance and distant metastasis in a spectrum of cancer types including liver. Emerging evidence shows that the tumor microenvironment plays a pivotal role for generating and harboring CSCs [22]. Stimuli and/or stress from the tumor microenvironment such as ROS burst from aggressive treatments and dysregulation of those ROS-dependent cellular processes are strongly associated with many cancers. However, the underlying mechanisms as how CSCs are generated remain poorly understood.

Previously, we demonstrated that FD induced OS and apoptosis in well-differentiated hepatocellular carcinoma (HCC) cells line HepG2 [23]. In this study, we used HCC as the main cell model (along with other cancer types including lung and glioma) to demonstrate that prolonged FD induced metastatic potential of cancer cells. Under FD culture condition, both SK-Hep1 and Mahlavu cells exhibited an increased intracellular oxidative/nitrosative stress (ONS) and decreased proliferation and colony forming ability. However, the metastatic abilities of HCC cells were markedly elevated, accompanied by the up-regulation of epithelial-mesenchymal-transition (EMT) gene products. More importantly, cancer stem-like phenotypes were promoted under FD condition. Finally, we provided *in vivo* evidence that tumor bearing mice fed with FD diet exhibited increased tumorigenic/metastatic potential when compared to their FC counterparts.

## RESULTS

### Folate deficiency (FD) induced oxidative-nitrosative stress (ONS) in HCC cells

**Figure 1:**
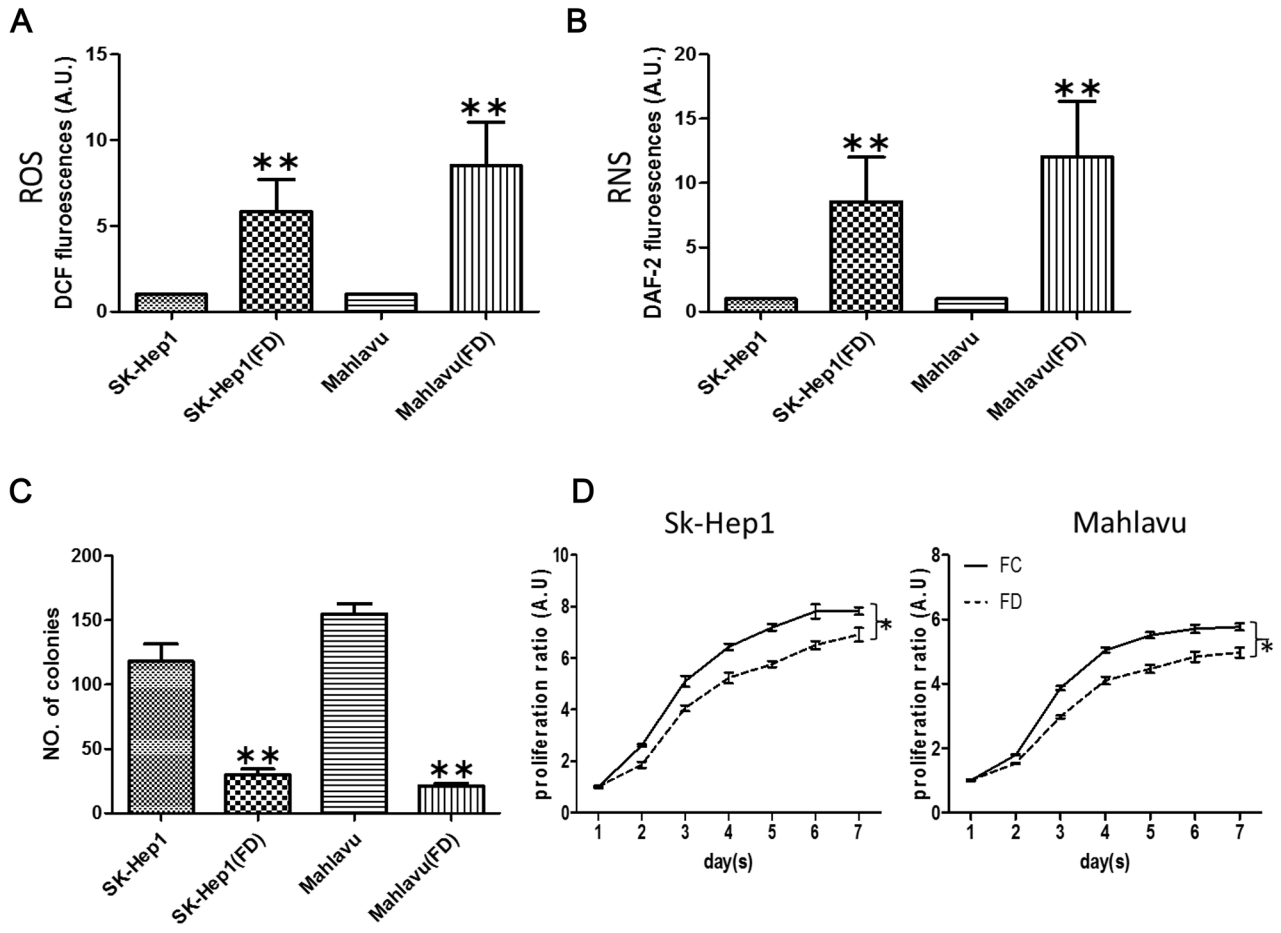
Folate deficiency promotes oxidative-nitrasive stress in HCC cell lines After two weeks of culture in folate deficient conditions, HCC cells SK-Hep1 and Mahlavu appeared to exhibit significant intracellular ROS **A.** and RNS **B.** respectively. The colony-forming **C.** and proliferative **D.** abilities were also lower. *p <0.05; **p<0.01 (N=3, compared to parental counterparts).

Sk-Hep1 and Mahlavu HCC cells were cultured under FC and FD conditions for different duration. Two weeks under FD condition, a significantly increased intracellular production of reactive oxygen (ROS, Figure 1A) and nitrogen species (RNS, Figure 1B) was observed using flow cytometry. When folate was re-supplemented, the intracellular ROS and RNS levels could be significantly suppressed, especially RNS (Data not shown). FD-conditioned cells exhibited a significantly lower colony-forming (Figure 1C) ability. Cellular proliferation was also decreased under FD condition (Figure 1D).

### FD promotes epithelial-to-mesenchymal transition (EMT)

**Figure 2:**
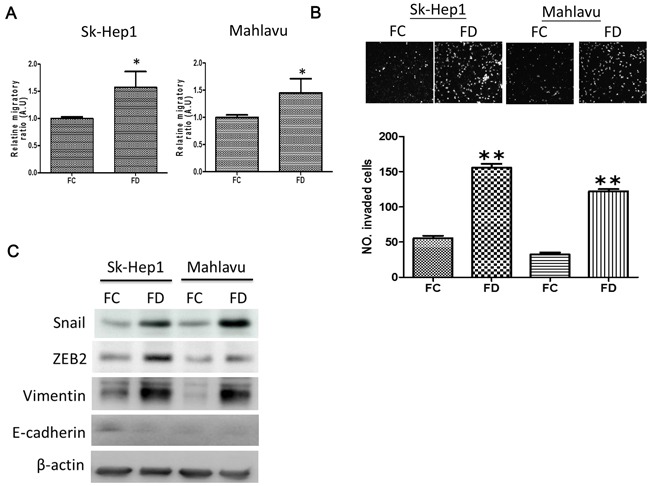
FD-conditioned HCC cells demonstrated enhanced metastatic ability **A.** Transwell analysis demonstrated FD-conditioned SK-Hep1 and Mahlavu cells exhibited a significantly higher migratory ability as compared to their parental counterparts. **B.** Metrigel invasive assay demonstrated that FD-conditioned HCC cells displayed a significantly higher invasive ability. **C.** Comparative EMT Western profiling. FD-conditioned SK-Hep1 and Mahlavu demonstrated an increased expression in mesenchymal markers such as Snail, ZEB2 and Vimentin. While epithelial marker, E-cadherin was undetectable. *p<0.05; **p<0.01.

We examined the effects of FD on the metastatic potential in cancer cells. FD-cultured Sk-Hep1 and Mahlavu cells exhibited heightened metastatic potential. For instance, FD-conditioned Sk-Hep1 and Mahlavu cells appeared to be approximately 2-fold and 1.5-fold more mobile than their FC-conditioned counterparts (Figure 2A, also Supplementary Figure S1) while approximately 4-fold more invasive (Figure 2B) respectively. Western blots analysis of FD cells demonstrated that mesenchymal markers including Snail, ZEB2 and Vimentin were up-regulated while epithelial marker, E-cadherin appeared to be un-detectable (Figure 2C).

### FD-conditioned cells exhibited cancer stem-like phenotype

**Figure 3:**
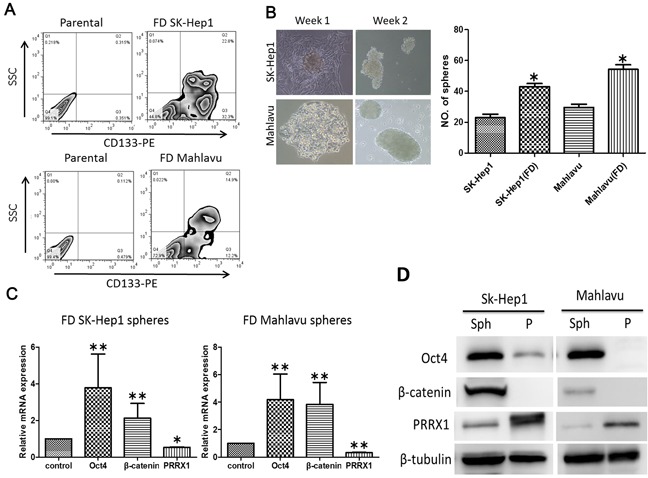
FD was associated with increased stemness in HCC cell lines **A.** Our FACS data demonstrated that both FD-conditioned HCC cells exhibited an increased percentage of CD133-positive cell populations. **B.** Under FD culture condition, both Sk-Hep1 and Mahlavu cells were able to generate a significantly higher number of tumor spheres. **C.** Q-PCR and Western **D.** analyses showed that spheres generated under FD conditions expressed a significantly higher mRNA and protein level of Oct4, β-catenin while decreased level of PRRX1. *p<0.05; **p<0.01.

Increased EMT potential has been shown to increase the generation of cancer stem-like cells [24]. Here, we observed an increased percentage of CD133-positive cells in FD-conditioned Mahlavu and Sk-Hep1 cells (approximately 55% and 27% respectively, Figure 3A; Supplementary Figure S1). Cell aggregates (or spheroids) started to emerge one week post FD-condition and more spheroids appeared while attached cells disappeared two weeks post FD-culture (inserts Figure 3B). Upon subsequent culture under serum-deprived condition, FD-conditioned Sk-Hep1 and Mahlavu cells were able to generate a higher number of spheres (Figure 3B; Supplementary Figure S1). Both mRNA (Figure 3C) and protein expression (Figure 3D) of the spheres formed under FD conditions showed increased stemness genes including Oct4, β-catenin while a decrease in PRRX1.

### FD-induced stemness was associated with down-regulation of miR-22

**Figure 4:**
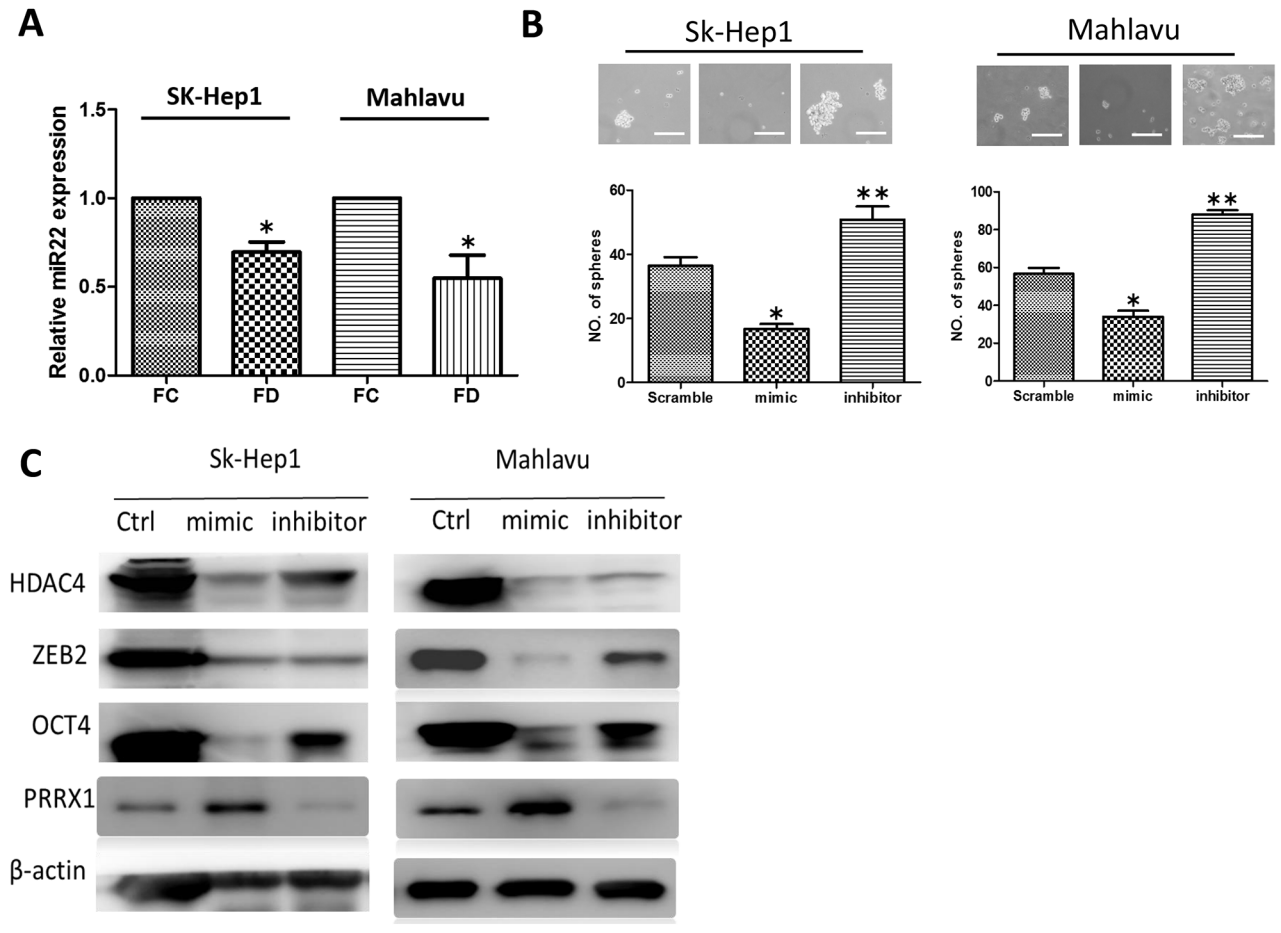
Down-regulation of miR-22 signaling axis in FD-conditioned HCC cells **A.** FD-conditioned Sk-Hep1 and Mahlavu cells appeared to express a significantly lower level of miR-22. **B.** The addition of miR-22 mimic significantly reduced the number of HCC spheres generated in both cell lines while the introduction of miR-22 inhibitor increased the number of spheres. **C.** Western blot analysis demonstrated that the expression of HDAC4, ZEB2, Oct4 was negatively correlated to the level of miR-22 while the reverse was true for PRRX1, in both cell lines.

MicroRNA-22 (miR-22) has been linked to c-Myc oncogenic pathway and shown to contribute to metastasis in breast cancer [25]. However, in hepatocellular carcinoma, a decreased level of miR-22 has recently been associated with poor prognosis in hepatoma patients [26]. Thus, we intended to examine the role of miR-22 in FD-conditioned HCC cell lines. We observed a significantly lower level of miR-22 in both FD-conditioned SK-Hep1 and Mahlavu cells (Figure 4A). When miR-22 expression was increased by exogenous mimic molecules, a significantly lower number of spheres generated from both Sk-Hep1 and Mahlavu cell lines while the reverse was observed when miR-22 inhibitor was added (Figure 4B). Subsequently, our Western blot analysis showed that miR-22 mimic treatment led to a decreased HDAC4, ZEB2 and Oct4 while an increased in PRRX1 and the reverse was observed when inhibitor was added (Figure 4C).

### FD-condition promoted metastatic potential in vivo

**Figure 5:**
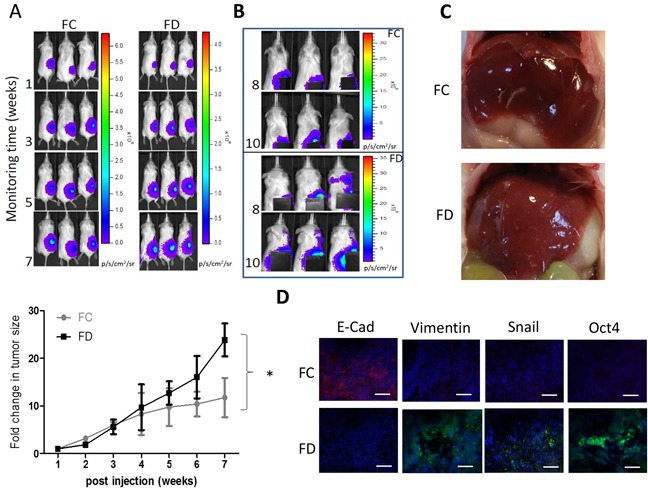
FD condition promoted Sk-Hep1 metastasis in vivo **A.** Sk-Hep1-bearing mice were subdivided into FC and FD groups. Tumorigenesis was observed using bioluminescence on a weekly basis. The fold change in tumor size was plotted against time. By the end of week 7 post tumor injection, FD group exhibited a significantly increased tumor growth rate as compared to FC counter parts. **B.** Starting from week 8 post tumor inoculation, metastasis was observed in FD animals. The primary tumor site was covered to demonstrate the metastatic signals. **C.** Representative images of the liver from FC and FD groups. Liver from FD animal showed multiple lesions (white spots) but not in FC animals. **D.** Immunofluorescence analysis of tumor biopsies demonstrating that lower intensity of E-cadherin and Oct4 in FD mice while high intensity of both Vimentin and Snail.

To validate our in vitro observations, firefly luciferase expressing Sk-Hep1 cells were subcutaneously injected in the right flanks of NOS/SCID mice. Mice were divided into two groups, folate complete (FC) diet and folate-deprived (FD) diet. *In vivo* SK-Hep1 tumorigenesis was monitored using bioluminescence imaging technique. For the first 2 weeks, the tumor growth rate in FD group appeared to be slightly slower (not significantly different, Figure 5A). However, a faster tumor growth rate became noticeable 5 weeks post injection and significantly different at week 7. Interestingly, starting from week 8 post tumor injection and folate deprivation, metastasis was detected in FD group (Figure 5B). Bioluminescent signal was detected away from the primary tumor site, appearing around the neck and head regions (Figure 5B, FD panel) but not in the FC mice. Lesions (white spots) were detected in the liver samples isolated from FD-treated mice while livers from FC mice exhibited no visible lesions (Figure 5C). Immunofluorescence analysis of the tumor biopsies from both group indicated mesenchymal markers Vimentin and Snail were higher in FD tumors and very weak E-cadherin signal; stemness marker Oct4 in FD tumor was also higher as compared to the FC counterpart (Figure 5D). A similar metastasis-promoting effects under FD condition was also demonstrated in mice bearing U87MG glioblastoma multiforme cells (Supplementary Figure S3).

## DISCUSSION

Tumor microenvironment plays a pivotal role in tumor progression and treatment failure in cancer patients. Recently, the emergence of the so-called cancer stem cells has also been associated to the tumor microenvironment [22]. One of the microenvironmental stress present in the cancer patient is micronutrient deficiency. Folate is an essential micronutrient which play a key role in the transfer of one-carbon moieties in processes including DNA synthesis, repair, and methylation. The usage of folate in cancer prevention and treatment has been extensively studied but remained controversial [27–30]. However, decreased serum folate concentration has been consistently observed in cancer patients and associated to advanced disease stage [5, 17, 31]. Ample evidence has indicated that folate deficiency contributes to DNA damage, genetic instability and tumor initiation [12, 13, 28], but its association to HCC tumorigenesis has been unclear. Here, we provided evidence that folate deprivation promoted metastatic potential and increased cancer stem-like properties via enhancing EMT process.

We first demonstrated that folate deprivation elicited a significant increase in both reactive oxygen and nitrogen species in both SK-Hep1 and Mahlavu cells; FD culture led to the suppressed proliferation and colony formation. Interestingly, under folate deprived condition, both cell lines remained viable but became dormant, evident by the resumption of proliferation upon folate replenishment (data not shown). This FD-induced dormancy suggested that HCC cells were capable of translating oxidative cues into a program of quiescence and behaving as dormant oxidant-resistant cells, as suggested by Pani et al [32]. In contrast, we previously demonstrated that the well-differentiated and least invasive HCC clonal variant HepG2 cells underwent apoptosis instead of dormancy under FD condition [23] while more malignant HCC cells such as SK-Hep1 and Mahlavu appeared to be more adaptive and acquired adaptation and drug resistance [33].

Recently, the role of oxidative stress, either within the tumor cells or from the surrounding stromal environment, has been re-examined and suggested as a key factor in promoting metastasis [34, 35]. It was hypothesized that metastasis represents an strategy for cancer cells to avoid oxidative stress-induced damage in the primary tumor site [32]. Among many strategies for which cancer cells acquired in face of the challenge of oxidative stress episode is redox adaptation, an emerging concept that explains the mechanisms by which cancer cells survive under ROS stress [36]. In agreement, FD-conditioned cancer cells (HCC, GBM and NSCLC cells) exhibited enhanced migratory and invasive abilities via undergoing EMT. Mesenchymal markers such as Snai1, ZEB2 and Vimentin were found up-regulated under FD condition while epithelial marker, E-cadherin was down-regulated. Furthermore, a previous study showed that folic acid acts to inhibit angiogenesis (inhibiting endothelial cell proliferation) through activating the cSrc/ERK 2/NF-kappaB/p53 pathway [37].

A seminal study has indicated that the ability to undergo EMT in breast cancer cells is associated with the increased generation of breast cancer stem cells [24]. In agreement, FD-conditioned HCC (Mahlavu and SK-Hep1), glioblastoma multiforme (U87MG) and lung cancer (H441) cells showed a significant increase in self-renewal ability as evident by the increased number of spheres formed and expression level of stemness associated markers such as Oct4, β-catenin and the percentage of CD133-positive cells. A recent study demonstrated that downregulation of paired related homeobox 1 (PRRX1) is associated with the acquisition of cancer stem cell (CSC)-like properties and poor prognosis in HCC patients [38]. In agreement, we also found that PRRX1 was down-regulated in the HCC spheres generated under FD condition.

Mechanistically, we have identified a negative association between miR-22 level and FD-induced EMT and stem cell properties. The functional and oncogenic role of miR-22 has been implicated as an epigenetic modifier and promoter of EMT and breast cancer stemness toward metastasis [25]. However, it appears that in HCC, miR-22 acts as a tumor suppressor. A recent study indicated that ectopic expression of miR-22 significantly inhibits HCC cell proliferation and tumourigenicity. More importantly, histone deacetylase 4 (HDAC4), known to have critical roles in cancer development, was shown to be directly targeted and regulated by miR-22 [26]. In concordance, ectopic miR-22 expression in both Sk-Hep1 and Mahlavu cells under FD conditions showed a significantly decreased self-renewal ability (less tumor spheres generated) while the reverse was observed with the addition of miR-22 inhibitor. These interesting observations strongly supports that the tumor microenvironment plays a pivotal role in the generation and maintenance of CSCs via a spectrum of molecular networks. Previously, an association between folate deficiency and the increased risk for developing colon cancer was suggested; sequence-specific alterations of DNA methylation in critical cancer-related genes might be involved in the folate deficiency-mediated colorectal carcinogenesis [39]. FD-conditioned HCC cells contained a lower level of miR-22 leading to the elevated level of HDAC4, an established oncogenic epigenetic modifier [40, 41], may provide a partial and mechanistic explanation for the association between cancer development and folate deficiency. Added support from a recent study indicated that vaproic acid, a histone deacetylase (HDAC) inhibitor, induced a decrease in HDAC4 and an increase in acetylated histone 4 (AcH4) and suppressed HCC cell growth [42].

Collectively, the increased EMT potential and expression of stemness genes under folate deprivation offered partial mechanistic explanation to the enhanced tumorigenesis in FD animals and the clinical observation where the serum folate status was correlated to cancer progression [17, 31].

## MATERIALS AND METHODS

### Reagents

Folate (pteroylmonoglutamic acid), amino acids, nucleosides, nucleotides and other chemical compounds were purchased form Sigma Chemical Co. (St. Louis, MO, USA). Minimal essential medium/alpha modified (αMEM) without ribosides, ribotides, deoxyriboside, deoxlribotides, glycine, serine and folate was specially ordered and formulated by JRH (Lenexa, KS, USA). Fetal bovine serum (FBS) was purchased from HyClone Laboratories (Logan, UT, USA). Penicillin, streptomycin, fungizone, trypsin and trypan blue were from GIBCO Laboratories (Grand Island, NY, USA).

### Cell lines and culture conditions

Human hepatocellular cell lines, SK-Hep1 and Mahlavu were obtained from the National Development Center of Biotechnology (Taipei, Taiwan) where U87MG (PTEN mutant, glioblastoma multiforme cell line) and non-small cell lung cancer cell line, H441 were obtained from ATCC. Cells were maintained as monolayer culture in complete medium at 37°C in a humidified 5% CO_2_ incubator with medium changed every 2 days. Complete medium contains αMEM with folate (2 μM), thymidine (36 μM), hypoxanthine (36 μM), glycine (600 μM), serine (250 μM) and 10% fetal bovine serem. Pencillin (20,000 units/L), streptomycin (20mg/L) and fungizone (2.5 mg/L) were also added. To formulate folate-deficient media, folate as well as thymidine, hypoxanthine and glycine were omitted from complete media to stress substrate availability in one-carbon metabolism. To minimize exogenous folate sources, fetal bovine serum was replaced with dialyzed fetal bovine serum (*d*FBS), which had been dialyzed at 4°C for 16 hours against 6×10 volumes of sterile PBS. Control medium was complete medium with 10% *d*FBS. Both cell lines cultured in folate-deficient medium (in the absence of folate and thymidine, hypoxanthine, glycine and serine) and complete medium were designed as folate-deficient (FD) cells and folate complete (FC) cells.

### Cell viability assay

Cancer cells under FC or FD conditions at various time intervals ranging from 24h, 48 h, 1 week and up to 2 weeks were seeded (1×10^3^) in 24-well microplates. Upon harvest, cell viability using SRB assay [43]. Briefly, upon harvest, cells were fixed with TCA (10%), followed by incubation at 4°C for 1 h, 3 washes and air-dry. Subsequently, cells were incubated at room temperature for 10 minutes in 100 μL of 0.4% (w/v) SRB prepared in 1% (v/v) acetic acid. The plates were then washed 4 times with 1% acetic acid and air-dried. The stained cells were incubated in 20 mmol/L Tris base (100 μL/well) at room temperature for 5 minutes. Optical densities were measured using a microplate reader (Molecular Devices, Sunnyvale, CA) at 562 nm.

### Colony formation assay

A total of 500 cells/plate were seeded onto six-well plates containing either FC or FD media and allowed to grow for 7 and 14 days. The plates were then stained with 5% crystal violet for 15 min, after which the cell colonies were counted, and photographed.

### Measurement of intracellular reactive oxygen and reactive nitrogen species

Intracellular productions of ROS, and NO were detected by flow cytometry using 2,7-chlorofluorescein diacetate (DCF-DA) and 4-amino-5-methylamino-2,7- dichlorofluorescein (DAF-2) as the probes, respectively. After 7- and 14-day of incubation, the culture medium was replaced with new medium containing 10 μM DCF-DA for 30 min in the dark at 37°C (for ROS) or 1 μM DAF-2 for 10 min in the dark (for RNS). The treated cells were washed once, and collected by centrifugation and re-suspended in PBS containing 20 μg/ml of propidium iodide (PI) for 5 min prior to flow cytometer. PI treatment differentiates between integrated and non-integrated cell membranes, since the latter permits the entrance of the dye into the cells, and the former does not. The DCF and DAF-2 fluorescences reflecting the intracellular ROS and NO in cells were measured by BD Accuri® C6 flow cytometer (BD Biosciences, Taipei, Taiwan).

### Invasion and migration assays

Invasion assay was performed according the protocol described previously [44]. For quantification, 3×10^5^ cells (treated with FC or FD condition previously) in serum free medium were seeded onto matrigel (Becton-Dickinson) in millicell culture plate inserts (pore size 30 mm, Millipore), three independent fields of invasion cells per well were photograph under phase-contrast microscopy. The number of cells per field was counted, and an average of the three determinations was obtained for each chamber. Each invasion assay was performed with a minimum of three times. For migration assay Transwell inserts (pore size 30 mm, Millipore) were used.

### Western blotting

Total cell lysates from different treatments were prepared using total protein cell lysis buffer containing protease inhibitor (Sigma). Protein concentration of lysates were determined by the Braford method. Samples were separated by 12% SDS-PAGE electrophoresis and transferred onto a PVDF membrane (Millipore). Nonspecific binding on the membrane was blocked with 0.1% Tween 20 in PBS containing 5% skim milk. Membranes were then incubated with primary antibodies overnight at 4°C, followed by incubating with appropriate secondary antibodies conjugated to horseradish peroxidase (HRP). Loading control was β-actin (Abcam). Membranes were subsequently washed and immunoreactive bands were detected using chemiluminescent HRP substrate (Millipore). Images were captured using BioSpectrum Imaging System (UVP, Upland, CA). Each Western blot was repeated to ensure reproducible results.

### Animal model and diets

The protocol of this study was approved by the institutional animal care committee of Taipei Medical University. Pellets of an L-amino acid-defined diet supplemented with 6.0 ppm or zero ppm folic acid diet were synthesized specially as the Laboratory Autoclavable Rodent Diet by LabDiet Co. (Brentwood, MO, USA). Folate deficient diet contained an antibiotic succinyl sulfothiazole (1.0%) to suppress folate production by the intestinal microflora. NOD/SCID mice (4-6 weeks of age) were purchased from BioLASCO Taiwan Co., Ltd (Taipei, Taiwan). After one day of fasting, the mice were randomly assigned to consume the FC or FD diets. They were pair-fed for an experimental period of 4 weeks with free access to water. Subsequently, the animals were injected with 1×10^7^ (in 100 μl of PBS) of SK-Hep1 cells which were previously cultivated in FC or FD medium for 2 weeks, into the right flank of the animal. *In vivo* bioluminescent images (BLI) was performed with IVIS 200 Imaging System (PerkinElmer). Images and measurements of bioluminescent signals were acquired and analyzed using Living Image Software (PerkinElmer). The change in tumor size was estimated by the fold change in BLI over time. Another set of experiment was performed using U87MG (human glioblastoma multiforme cell line) with the same protocol.

### Immunofluorescence

Upon sacrifice, Sk-Hep1 tumor cells were harvested for immunofluorescence. After washing twice with PBS, the cells were fixed in paraformaldehyde in PBS, permeabilized with 0.1% Triton X-100, and blocked with 1% BSA in PBS for 1 h. The dishes were first incubated with the indicated antibodies for 1 h, washed twice with PBS, and then incubated with Alexa 488-phalloidin solution (1:40) and the corresponding FITC-conjugated secondary antibodies for 30 min in the dark. Cell nuclei were dyed with DAPI (Vector Laboratories, Burlingame, CA). After washing, the slides were treated with an anti-fade reagent to prevent quenching of the fluorophores, and the cells were visualized using an A1R-A1 confocal laser microscope system (Nikon, Tokyo, Japan).

### Statistical analysis

The data are reported as mean ± SD. A value of **p < 0.01 was considered significant.

## CONCLUSION

Our findings provided new insight into the role of micronutrient folate in relation to cancer progression and generation of CSCs. FD, either as the result of dysregulated metabolism within the tumor or chemotherapies, creates a microenvironment where oxidative-nitrosative stress serves as the underpinning to initiate EMT and necessary stimuli for generating CSCs. Although the complete profile of molecular networks altered under FD in the three cancer types (HCC, GBM and NSCLC) examined in this study requires further elucidation, monitoring and manipulating folate status in cancer patients may represent an important venue to improve treatment efficacy and prevention of distant metastasis.

## SUPPLEMENTARY FIGURES


