# Supplementary material for: Folate deficient tumor microenvironment promotes epithelial-to-mesenchymal transition and cancer stem-like phenotypes
Source: Oncotarget. 2016 Apr 22;7(22):33246–56. doi: 10.18632/oncotarget.8910 (PMC5078091; doi:10.18632/oncotarget.8910)
Supplement: Supplementary file 1 [file oncotarget-07-33246-s001.pdf]

SUPPLEMENTARY FIGURES

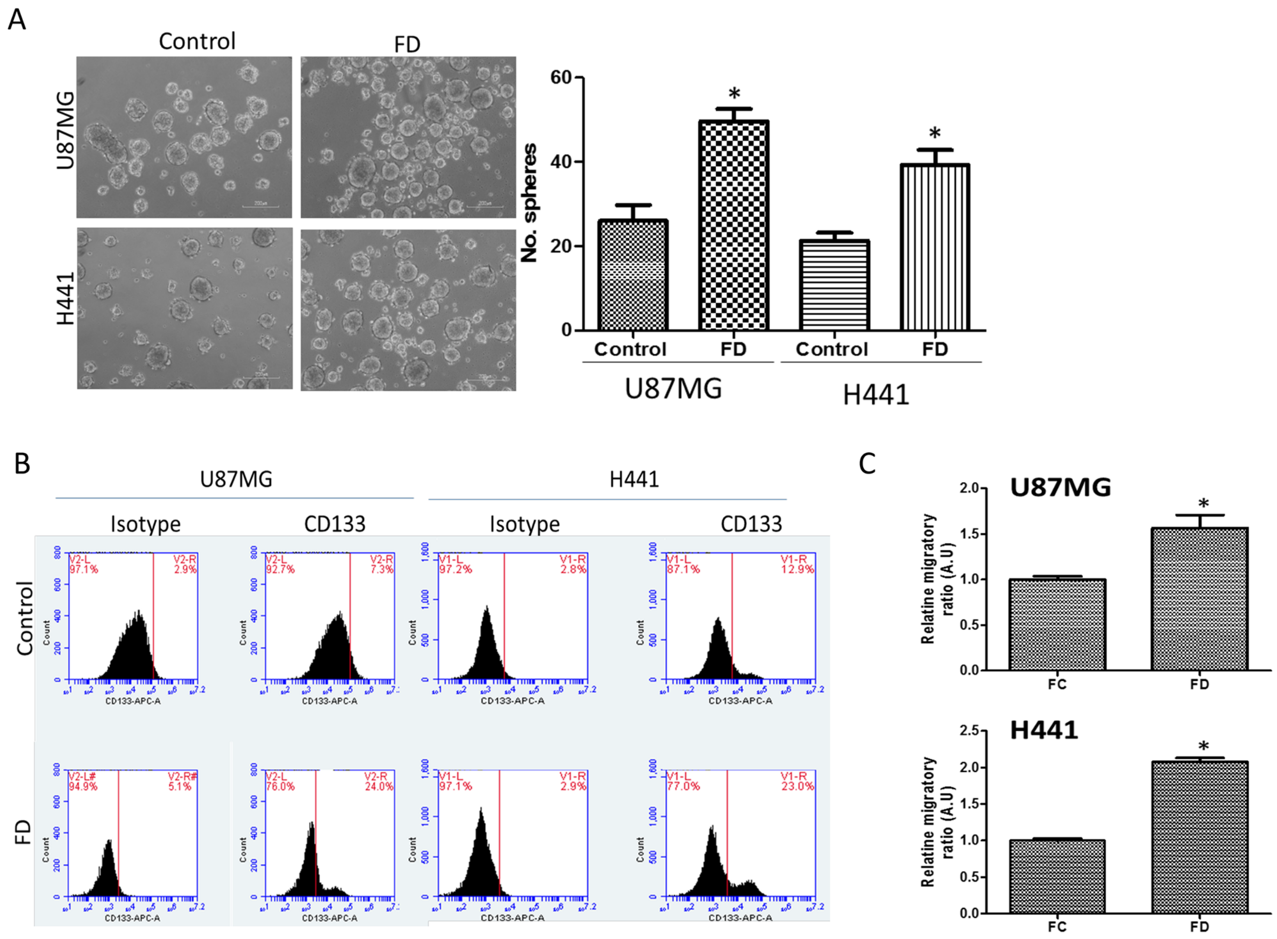

Supplementary Figure S1: FD condition promotes the generation of tumor spheres in both glioma and lung cancer cell lines. A. Glioma cell line. U87MG mPTEN and NSCLC cell line, H441 were cultured in both control and folate-depleted (FD) conditions for two weeks. Under FD condition, both U87MG mPTEN and H441 cell lines generated a significantly more number of tumor spheres as compared to those in control, folate-containing medium. **B.** Flow cytometric analysis demonstrated that under FD condition, the percentage of CD133+ U87MG and H441 cells was increased. **C.** Increased migratory ability was observed in FD U87MG and H441 cells as compared to their FC (folate complete) counterparts. *$p<0.05$.

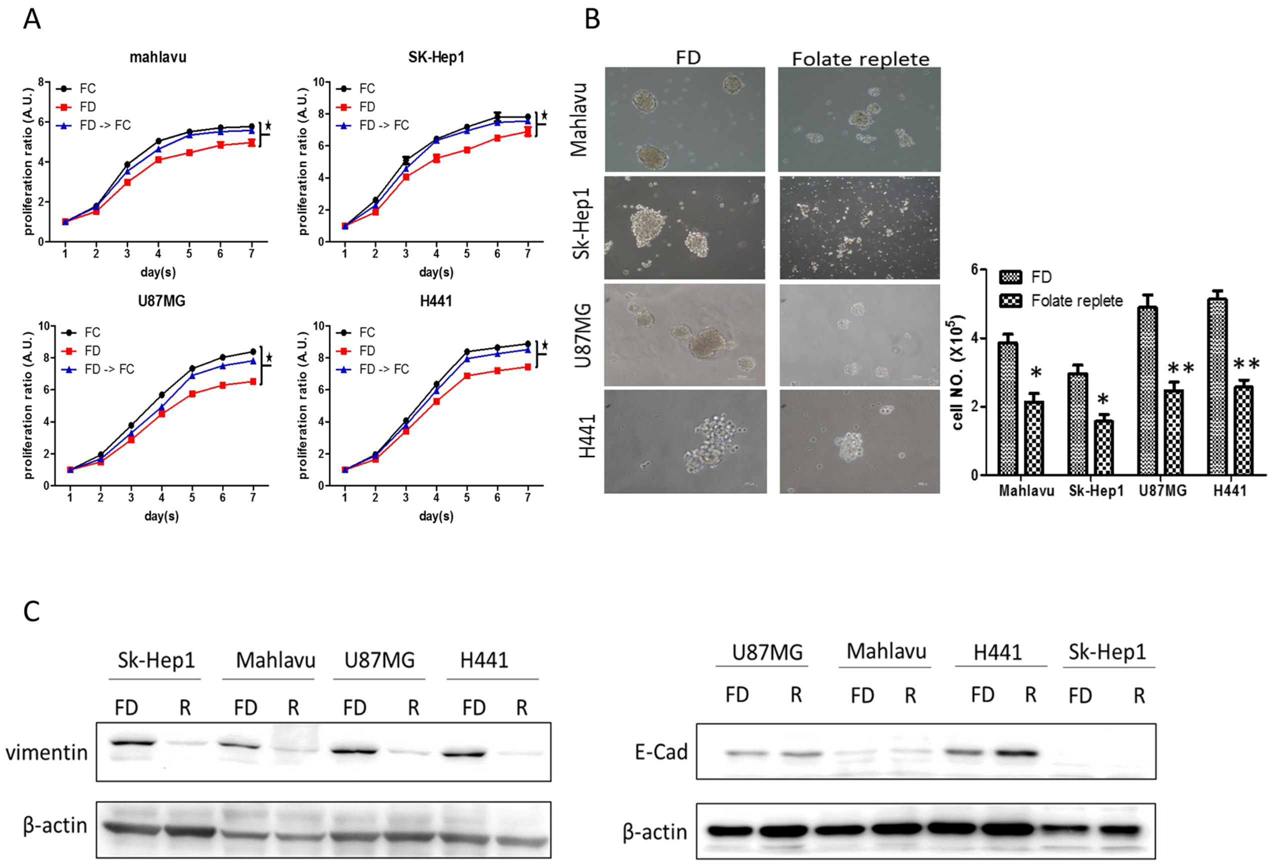

Supplementary Figure S2: Folate repletion induced reversal of FD-associated EMT and increased stemness. A. SRB analysis demonstrated that the reduced proliferative ability was almost completed regained upon folate repletion all three cancer types examined. **B.** Upon the repletion of folate, the sphere-forming ability appeared to be decreased as demonstrated by the reduced number of tumor spheres generated. *$p<0.05$; **$p<0.01$. **C.** Western blot analysis showed that upon folate repletion (R), epithelial marker (E-cadherin) was increased while mesenchymal marker (vimentin) was decreased.

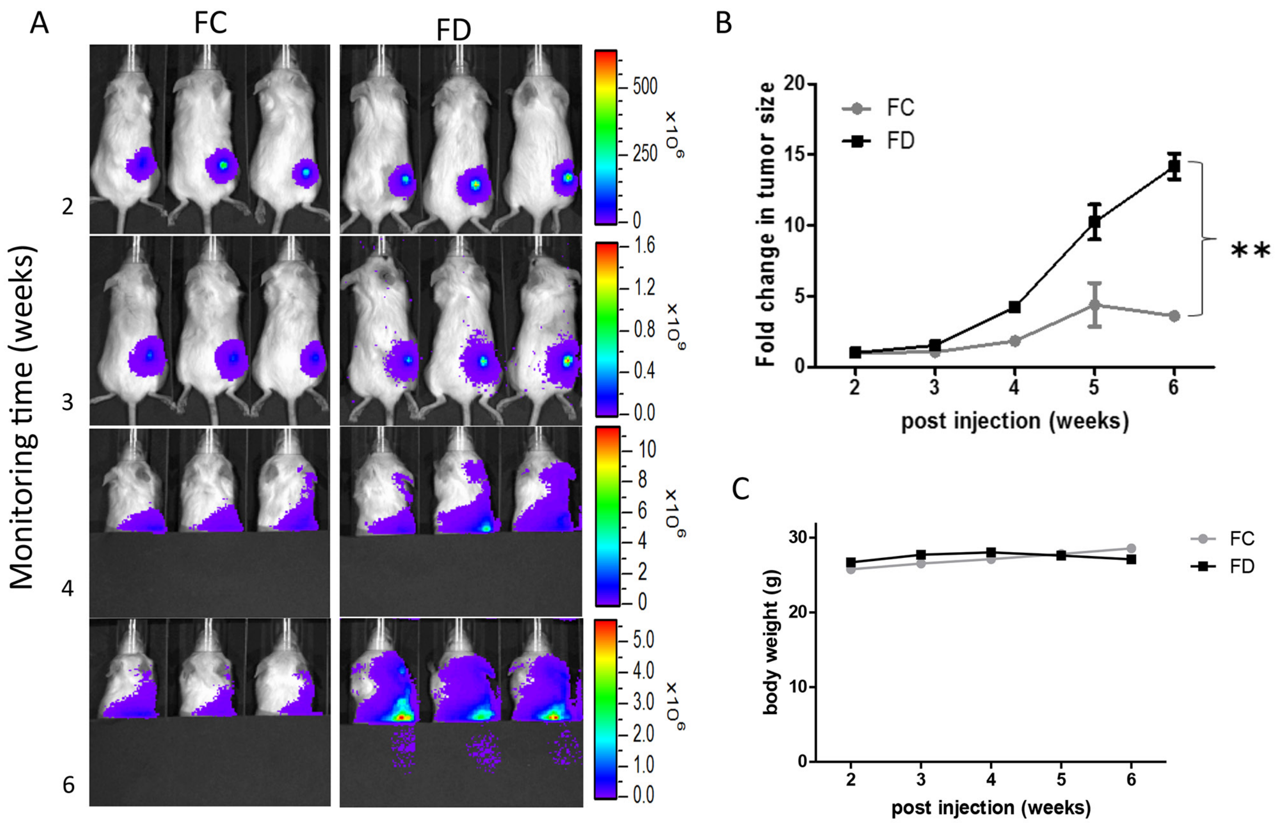

Supplementary Figure S3: Folate deficiency induced distant metastasis of GBM cells, U87MG in vivo. A. Non-invasive bioluminescence imaging showed that a higher and distant metastasis was detected earlier in the U87MG-bearing mice under FD diet as compared to their FC (folate complete) counterparts. **B.** Semi-quantitative analysis of bioluminescence. The change in tumor size was represented by the fold-change in bioluminescence over time. FD group showed a significantly higher tumor burden. ** $P<0.01$. **C.** The bodyweight of both FD and FC mice were measured and tracked weekly. There was no apparent difference in bodyweight.
